# Conversion of chemical to mechanical energy by the nucleotide binding domains of ABCB1

**DOI:** 10.1038/s41598-020-59403-7

**Published:** 2020-02-13

**Authors:** Dániel Szöllősi, Peter Chiba, Gergely Szakacs, Thomas Stockner

**Affiliations:** 1 Medical University of Vienna, Center for Physiology and Pharmacology, Institute of Pharmacology, Waehringerstr. 13A, 1090 Vienna, Austria; 2 Medical University of Vienna, Institute of Medical Chemistry, Center for Pathobiochemistry and Genetics, Waehringerstr. 10, 1090 Vienna, Austria; 3 Medical University of Vienna, Institute of Cancer Research, Borschkegasse 8A, 1090 Vienna, Austria

**Keywords:** Transporters, Molecular modelling

## Abstract

P-glycoprotein (ABCB1) is an important component of barrier tissues that extrudes a wide range of chemically unrelated compounds. ABCB1 consists of two transmembrane domains forming the substrate binding and translocation domain, and of two cytoplasmic nucleotide binding domains (NBDs) that provide the energy by binding and hydrolyzing ATP. We analyzed the mechanistic and energetic properties of the NBD dimer via molecular dynamics simulations. We find that MgATP stabilizes the NBD dimer through strong attractive forces by serving as an interaction hub. The irreversible ATP hydrolysis step converts the chemical energy stored in the phosphate bonds of ATP into potential energy. Following ATP hydrolysis, interactions between the NBDs and the ATP hydrolysis products MgADP + P_i_ remain strong, mainly because Mg^2+^ forms stabilizing interactions with ADP and P_i_. Despite these stabilizing interactions MgADP + P_i_ are unable to hold the dimer together, which becomes separated by avid interactions of MgADP + P_i_ with water. ATP binding to the open NBDs and ATP hydrolysis in the closed NBD dimer represent two steps of energy input, each leading to the formation of a high energy state. Relaxation from these high energy states occurs through conformational changes that push ABCB1 through the transport cycle.

## Introduction

ABCB1 (P-glycoprotein) is responsible for multidrug resistance in cancer cells by preventing drugs from reaching the cytosol, thereby leading to failure of chemotherapy treatment^1^. Human ABCB1 is a major player in drug disposition and pharmacokinetics of many prescription drugs^2^. It is expressed at barrier tissues such as the blood-brain-barrier, gastrointestinal tract, placenta, ovary and testis, but also in liver and kidney^3–5^. ABCB1 is an archetypical ABC transporter, consisting of two transmembrane domains (TMDs) and two nucleotide binding domains (NBDs). The TMD dimer encompasses the substrate translocation path, which recognizes and expels numerous unrelated compounds^6^. The NBDs, the most conserved region of ABC proteins, form at their interface two symmetry-related nucleotide binding sites (NBSs) that provide the energy for substrate transport through ATP binding and hydrolysis. Despite the remarkable amount of work on ABCB1 (for reviews see^7–12^), the molecular mechanism of substrate transport is not fully understood. In particular, the exact conformations along the transport cycle, the number of ATPs hydrolyzed per transported substrate and the conformational consequences of ATP hydrolysis remain controversial^9^.

The role of conserved motifs in the NBDs for MgATP interactions and interdomain communication have been described^11,13^. These important motifs are the A-loop^14^, the Walker A and B motifs^15^, the H-loop^16,17^ and the Q-loop^18^ from one NBD and the D-loop^19,20^, X-loop^21^ and signature motif ^20^ from the other NBD. The A-loop and the Walker A motif have a major role in nucleotide binding, the Walker B motif and the H-loop^22^ play a direct role in ATP hydrolysis, and the Q-loop^23^, X-loop^21^ and D-loop^24^ are responsible for interdomain communication. Biochemical and structural data have identified conformational changes of the NBD dimer in response to ATP binding and hydrolysis. Association and stabilization of the NBD dimer in response to ATP binding was shown by several methods, most prominently cryo-EM^25,26^, EPR^27,28^, FRET^29^, electron microscopy^30^, cysteine cross-linking^31^, and mutation studies^32^.

ATP and ADP bind to ABCB1 with a K_D_ of 0.28 mM and 0.33 mM, respectively^33^, whereby the intracellular concentration of ADP is comparable to its K_D_, while ATP is present in over 10-fold excess. Binding of MgATP induces NBD dimerisation^34,35^. We have previously shown that interactions with ATP provide the energy for NBD dimerisation^36^. Elucidation of the driving forces that induce conformational changes and the energy barriers separating states is required for a full understanding of the mechanism by which ATP energizes the transport cycle of ABCB1. Our aim was to characterize the process by which the chemical energy stored in the phosphate bonds of ATP is converted into mechanical forces that allow ABCB1 to advance though the transport cycle. Free energy profiles and the underlying forces were derived using molecular dynamics (MD) simulations in combination with umbrella sampling to determine Potential of Mean Force (PMF) profiles using a model of isolated NBDs. PMF profiles allow for quantifying the energy output produced by the NBDs, revealing the maximum energy that can be harvested by ABCB1 for substrate transport.

## Materials and Methods

### Model creation

Models of the NBDs of ABCB1 were created and simulations were carried out as described in Szöllősi *et al*.^36^. In brief, ABCB1 was modeled based on the structure of Sav1866^21^ using Modeller version 9.15^37,38^, and the NBDs (residues 390–620 and 1033–1265) were extracted from the full-length model. Supplementary Fig. 1 shows the sequence alignment^39^ of NBD1 and NBD2 of ABCB1 with the NBD of Sav1866. The sequence identities of Sav1866 with NBD1 and NBD2 of ABCB1 are 51% and 48%, respectively, indicating that very reliable models can be created. Sav1866 was preferred over CmABCB1 as template despite the marginally higher sequence similarity of CmABCB1^40^ with the NBDs of ABCB1 (56% and 52% for NBD1 and NBD2, respectively), because 2 mutations were introduced in CmABCB1 to allow for crystallization, no ADP or ATP bound structure exists, the catalytic glutamate is in an unusual conformation and the inward facing conformation shows extensive structural changes of the functionally important Walker A and Walker B motifs and the D-loop. Sav1866 was also preferred over the outward facing cryo-EM structure of human ABCB1^25^, as this structure at 3.4 Å did not reach atomic resolution and shows rotations of the core domain relative to the scaffold, leading to displacements of the Walker A motif relative to the signature sequence. These structural distortions lead to non-canonical geometries of the NBSs, which might be a consequence of the catalytic glutamate mutation in the Walker B sequences. Models of human ABCB1 were generated using the automodel procedure of Modeller, version 9.15, and ranked by the DOPE score. The best model was selected for simulations. Nucleotides were placed into the nucleotide binding sites (NBSs) as observed for AMP-PNP in Sav1866. In the post-hydrolytic state (ADP + P_i_), the P_i_ group was placed closest (without atom overlap with ADP) to the position of the Pγ of MgATP.

### Reaction coordinate for potential of mean force calculations

**Figure 1:**
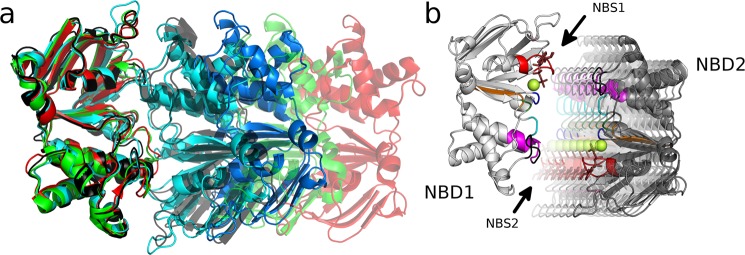
Definition of the reaction coordinate. (**a**) Panel A shows an overlay of the NBDs of mouse (PDB ID: 4M1M, green), C. elegans ABCB1 (PDB ID: 4F4C, red) and human ABCB1 (PDB ID: 6C0V, black) with the starting structure at the minimal (cyan) and at 1.4 nm NBD separation (blue), after fitting all structures to NBD1. The displacement of NBD2 shows the movements observed in the respective structures. (**b**) Structural models of the simulated NBD dimer. Transparent structures indicate the mode of NBD separation used for potential of mean force (PMF) simulations. Conserved motifs are highlighted: ATP (brown sticks), Mg^2+^ (light green), A loop (pink), Walker A (red), Q loop (green), Walker B (orange), H loop (blue), X loop (black), signature motif (magenta) and D loop (cyan).

When positioning the NBDs along the reaction coordinate used for the Potential of Mean Force (PMF) calculations, the P_i_ stays attached to the signature motif, while the nucleotides (ATP and ADP) remain in contact with the A-loop and the Walker A motif, as these loops were experimentally found to interact with nucleotides in isolated NBDs^41^. The reaction coordinate represents the separation of the NBDs along a single axis that was derived from crystallographic data, which revealed NBD movements of full-length transporters (Fig. 1). Large rotations or translations are restricted by the TMDs, restraining motions of the NBDs in the directions orthogonal to the reaction coordinate: i) motions normal to the membrane plain (along the Z axis) are prevented by the transporter structure; ii) motions along the Y axis are strongly restrained as each NBD is attached to the coupling helices of the TMD domains. The helices of these intracellular loops (ICLs) are extensions of the transmembrane helices that form two stable tetrahedral bundles (TMH2,3,10,11) and (TMH4,5,8,9), which restrain bending, while the large TMD-TMD interface in the transmembrane region stabilizes the relative orientation of the TMDs. iii) The extent of rotational motions is also limited by the TMDs and the ICLs, rigidified by the tetrahedral bundles and two interacting coupling helices per NBD. Crystal structures show that the NBD-TMD interface remains intact and rotation of the two NBDs relative to each other are very small. Exceptions come from extensive crystal contacts or stem from a non-functional degenerate NBS (e.g. the heterodimeric TM287/288 transport^42^), for which it remains uncertain if the mechanism of transport is the same. A rotation of a few degrees or a movement normal to the tested reaction coordinate of NBD separation may not *a priori* be excluded, but if larger than a few degrees, it would also require non-linear or disruptive changes in TMD conformation.

Crystal structures of ABC transporters, in particular structures of human ABCB1 and the highly homologous mouse ABCB1 and C. elegans ABCB1, represent snapshots of the motion from the closed dimer (outward-facing, ATP bound) to the inward-facing nucleotide free conformation (Fig. 1a). We defined a single dimensional reaction coordinate of NBD separation that aligns with a motion which is symmetric with respect to NBS1 and NBS2 (Fig. 1b) to achieve maximal overlap with the path connecting cryo-EM and crystal structures. Exceptions are the structures of human ABCB1 with the PDB ID: 6QEX^26^ and of CmABCB1 with the PDB ID: 6A6M^40^. The deviations from the symmetric reaction coordinate are most likely caused by the asymmetric distortions and unfolding of transmembrane helices, which in case of the CmABCB1 structure also lead to unfolding of the ATP interacting loops in the NBDs.

### Potential of mean force calculations

Potential of Mean Force (PMF) calculations allow for deriving the free energy profile along a restrained reaction coordinate provided all other degrees of freedom are equilibrated. The PMF profile therefore reports on how the free energy changes along the reaction coordinate of increasing distance between the NBDs. We derive PMF profiles by using umbrella sampling, which consist of a large set of independent simulations that record the energy needed to hold the NBDs in place with respect to the reaction coordinate. Each umbrella simulation was 10 ns long, which is sufficient for equilibration and convergence. The PMF profiles were derived by applying the weighted histogram analysis method (WHAM)^43,44^.

Starting configurations were created by positioning the NBDs, including the attached nucleotide, P_i_ and Mg^2+^, if present, relative to each other, along the reaction coordinate in 0.04 nm steps until reaching a maximal separation of 1.4 nm. Additional windows in 0.01 nm steps were added between 0.12 and 0.48 nm to enhance sampling. During PMF simulations, the distance between the NBDs was maintained by applying the umbrella potential to two groups defined as each consisting of the Cα atoms of one NBD, the attached nucleotide, Mg^2+^ and P_i_ (if present). The relative alignment of NBDs was maintained using the Enforced Rotation (ER) approach^45^. ER was applied independently to both groups and separately to each rotation axis. The force constant used for the umbrella potential was 5000 kJ/mol/nm^2^, while applying a force constant of 1000 kJ/mol/nm^2^ to maintain the alignment using ER. The apo state was simulated in the absence of nucleotides, the pre-hydrolytic state in the presence of MgATP, while the post-hydrolytic state was simulated in the presence of the hydrolysis products MgADP and P_i_ (HPO_4_^2−^ or H_2_PO_4_^−^). Following hydrolysis, the protonation state of P_i_ is uncertain as either HPO_4_^2−^ or H_2_PO_4_^−^ could be present^46^. According to a hybrid quantum mechanical/molecular mechanical simulation^47^, HPO_4_^2−^ and H_2_PO_4_^−^ are present in an equilibrium in the NBSs and show a population ratio of 1:3, separated by a small transition energy barrier of ~2.5 kJ/mol. Simulations were carried out with Gromacs 5.1.4^48^ using the Amber99sb-ildn force field^49^. ATP and ADP parameters are based on the GAFF force field^50^ as parameterized by Meagher *et al*.^51^. Original parameters were converted to the Gromacs format using acpype^52^. PMF profiles were derived from these trajectories by applying the weighted histogram analysis method (WHAM)^43,44^ using 10 ns long biased simulations after discarding the first 0.5 ns as equilibration.

Temperature was maintained at 310 K using the v-rescale (τ = 0.1 ps) thermostat^53^, while separately coupling protein and solvent. Pressure was maintained at 1 bar using the Parrinello-Rahman barostat^54^ applying a coupling constant of 1.0 ps. Long range electrostatic interactions were described using the smooth particle mesh Ewald method^55^ applying a cutoff of 1.0 nm. The van der Waals interactions were described using the Lennard Jones potentials applying a cutoff of 1.0 nm. Long range corrections for energy and pressure were applied. The complete set of parameters used for the simulations are listed in the Supplementary Information.

## Results

Energetic contributions to the transport cycle of ABCB1 are manifold, including substrate binding and release, ATP binding, ATP hydrolysis, ADP and P_i_ release, conformational changes, lateral movements against the membrane lipids, water entering and leaving the NBD and TMD interfaces. The energetic profile is therefore convoluted by several factors, and the majority of the energy output of the NBDs is immediately consumed by the TMDs. We created an isolated NBD system, which allows for separating the energetic contributions of the NBDs from those of the TMDs with the aim to quantify the maximal energy output produced by the NBDs and derive the free energy profile associated with NBD movement. These data provide the framework for understanding how energy is converted from the chemical energy stored in the phosphate bonds of ATP into motions leading to substrate translocation by proceeding through conformational states of the transport cycle.

**Figure 2:**
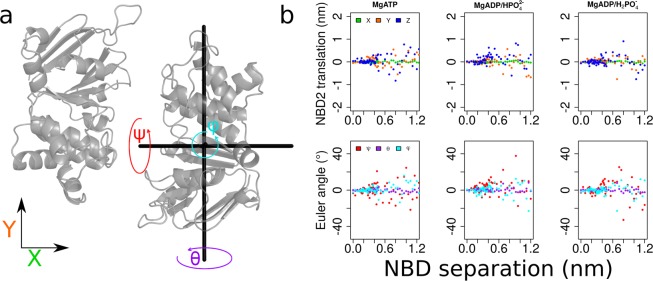
Unrestrained simulations confirm the absence of potential artifacts. Each umbrella simulation was extended by 1 ns without the ER restraints, while holding NBD1 in place by a position restraint (only the distance between the NBDs was maintained to avoid re-association). (**a**) Structure of the NBD dimer that serves as structural legend to display the directions of translational and rotational movements (color coded) as used in panel b. The XY plane is oriented parallel to the membrane. (**b**) The upper row shows translational movements of NBD2 relative to its starting position. The lower row shows the rotations of NBD2 relative to its starting alignment. Each data point shows values computed at the end of the 1 ns extension simulations.

We use PMF calculations to quantify the energetic profile along the predefined reaction coordinate of NBD separation, which coincides with NBD motions observed in transporter structures (Fig. 1). We extended every 10 ns long umbrella simulation by 1 ns to unmask potential artifacts induced by the simulation setup, as we used restraints to prevent uncontrolled rotations and translations. The procedure, described in detail in Szöllősi *et al*.^36^ was applied. In a nutshell, all restraints were removed, except the distance restraint needed to maintain NBD-NBD separation. Unmasked artifacts would lead to systematic deviations consistently pointing into the same direction. Analysis of the 1 ns long extensions (Fig. 2) shows no such systematic deviation from the starting geometry, suggesting that deviations were random. This indicates that the reaction coordinate is free of hidden high energy conformations, which could alter the PMF profiles. The only exception might be the ψ-angle that shows a tendency for rotation at intermediate distance (at 0.4–0.6 nm), indicating that the NBDs might rotate up to 10° relative to each other.

**Figure 3:**
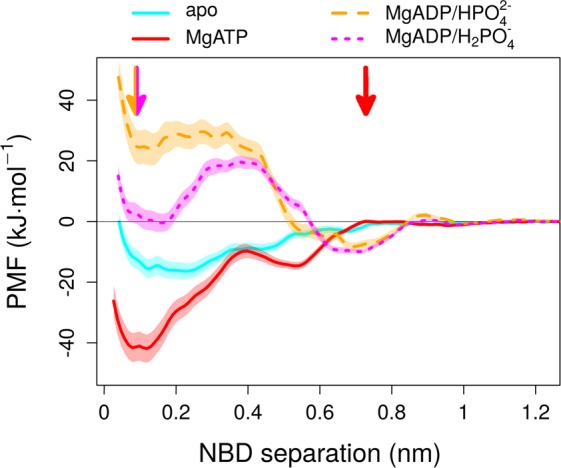
Potential of Mean Force (PMF) profiles as a function of NBD separation: Nucleotide dependent PMF profiles of NBD separation. The arrows indicate initial high energy conformations. Error bars were derived by the bootstrapping method.

The interaction energy is zero for non-interacting NBDs at large to infinite distance. A profile rising from negative values at short distances towards zero at large distances reflects a stable dimer, while a decaying profile indicates that the closed dimer is of high energy. Strictly speaking, only the shape of the four profiles can be directly compared, but not the absolute values, because the chemical potential μ of the NBDs at infinite distance differs between systems. The chemical potential μ is not the same, because the NBDs are ligand free or interacting with MgATP, MgADP·HPO_4_^2−^ or MgADP·H_2_PO_4_^−^. The apo system (Fig. 3, cyan line) shows a shallow minimum in the PMF profile, which is indicative of a weakly interacting dimer^36^, in line with experiments showing that isolated NBDs of ABC transporters dimerize in a nucleotide dependent manner^56,57^. We find that the presence of MgATP bound to the NBSs (Fig. 3, red) leads to a dramatic change in the PMF profile. The MgATP-bound NBD dimer shows a deep energy minimum, which is stabilized by −41.8 ± 4.6 kJ/mol relative to the fully separated nucleotide-bound NBDs^58^. The products of ATP hydrolysis are MgADP and P_i_. The pKa values of P_i_ are 2.1, 7.2, and 12.3, thus the protonation state of P_i_ is uncertain at physiological pH, as P_i_ could be single (HPO_4_^2−^) or double protonated (H_2_PO_4_^−^). We therefore calculated the PMF profiles for MgADP·HPO_4_^2−^ and MgADP·H_2_PO_4_^−^, even though highly charged environments such as the NBSs of ABC transporters favor the higher charged form HPO_4_^2−^. We find that the presence of the ATP hydrolysis products MgADP and HPO_4_^2−^ (orange) converts the stable MgATP bound pre-hydrolysis state into a high energy state that favors NBD dissociation by (+24.4 ± 4.7 kJ/mol). Interestingly, the PMF profile shows an initial phase (NBD-NBD separation <0.35 nm) with a flat profile, indicative of a stochastic onset of dimer opening. The initial steep slope close to zero, visible in all four profiles, is caused by atomic overlaps once NBD1 and NBD2 come too close. However, a steep energy gradient drives NBD movements beyond a separation of 0.35 nm. The MgADP·H_2_PO_4_^−^ bound NBD dimer (magenta) is also unstable as compared to the fully separated NBDs, but the PMF profile also reveals a shape that includes an energy barrier of +19.7 ± 2.0 kJ/mol at ~0.35 nm, which prevents dissociation beyond 0.2 nm. However, due to the low barrier^47^ separating single and double protonated P_i_, this barrier in the PMF profile can be circumvented by deprotonation to HPO_4_^2−^. Consistently, experimental data for Chinese hamster ABCB1 showed that a pH below 6.5 blocks ATPase activity^59^.

**Figure 4:**
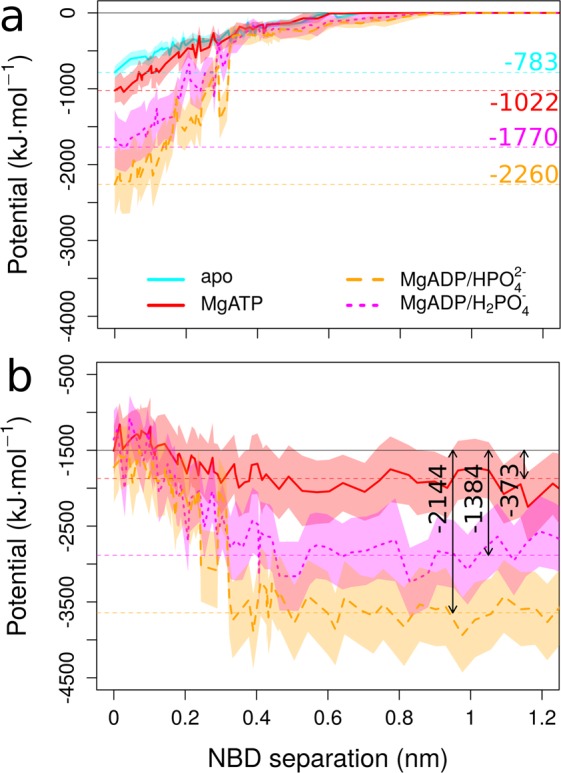
Enthalpic contributions to the PMF. (**a**) Sum of interaction potentials (electrostatic + van der Waals) between NBDs and bound nucleotide, P_i_, and Mg^2+^ (if present). (**b**) Sum of enthalpic interaction between nucleotide, P_i_, Mg^2+^ and the solvent of the MgATP, MgADP·HPO_4_^2−^ or MgADP·H_2_PO_4_^−^ bound NBDs. Error bars represent standard deviations of individual umbrella windows.

Next, we investigated individual contributions to the PMF profiles to identify dominant forces that induce NBD separation after ATP hydrolysis. Analyses of the interactions between the separating NBDs, including bound Mg^2+^ + nucleotide + P_i_ (if present), show the smallest enthalpic interactions (Fig. 4a) for the apo system. The strength of interactions increases with the presence of MgATP, but the attractive forces are largest for MgADP·HPO_4_^2−^ and MgADP·H_2_PO_4_^−^. Their increased strength is facilitated by Mg^2+^, which bridges ADP and P_i_ at separations below 0.4 nm. Thereby, the double charge of Mg^2+^ counteracts the repulsion between ADP and P_i_. The Mg^2+^ to HPO_4_^2−^ interaction contributes −670 ± 72 kJ/mol per NBS, while the Mg^2+^ to ADP interaction reaches −711 ± 46 kJ/mol per NBS. The cumulative forces are attractive for the MgADP·P_i_ containing systems and can therefore not explain the repulsion observed in the PMF profile of the post-hydrolytic state (Fig. 3).

Next, we considered interactions of Mg^2+^ + nucleotide + P_i_ with the solvent as increasing NBD separation leads to the presence of more water molecules in the NBD interface. Figure 4b shows the total interaction energies of Mg^2+^ + nucleotide + P_i_ with water as a function of NBD separation. The interaction energy of MgATP with water increases only moderately with NBD separation and reaches approximately 30% of the NBD-NBD interactions, indicating that the stronger NBD-NBD interactions stabilize the ATP-bound dimer (Fig. 4, cyan traces). In contrast to the pre-hydrolytic MgATP bound state, the interaction energy of MgADP·H_2_PO_4_^−^ with water reaches 80% of the enthalpic energy cost for separating the NBDs (Fig. 4, magenta traces). For the MgADP·HPO_4_^2−^ bound state, the gain in interaction energy with water equals the loss in interactions between the MgADP·HPO_4_^2−^ bound NBDs (Fig. 4, orange traces). Thus, solvation of Mg^2+^ + nucleotide + P_i_ makes a major energetic contribution to the PMF, leading to NBD separation in the post-hydrolytic state.

**Figure 5:**
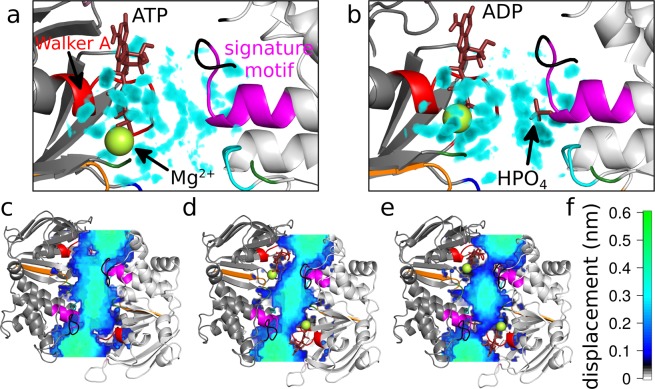
Water density reveals structural water molecules in the NBS. Density of water molecules in NBS1 is shown as density map (cyan), if its density is two times above normal water density. These islets indicate stably bound water molecules around the phosphate groups, (**a**) of MgATP and (**b**) of MgADP·HPO_4_^2−^, at 0.72 nm NBD separation. NBD motifs, nucleotides and Mg^2+^ are colored as in Fig. 1. Water displacement for (**c**) apo, (**d**) MgATP and (**e**) MgADP·HPO_4_^2−^ shown in a slice positioned at the level of the γP. (**f**) Color scale for water displacement (for comparison; displacement of unrestrained water molecules is ~0.55 nm).

To further investigate water properties, we determined water density by calculating average water positions for each umbrella window. Bound or structural water molecules do not move and become visible as water sized voxels of high density (see density map on Fig. 5). These small islets are separated from the diffusing solvent or other bound water molecules due to their immobility and the finite size of the individual stably bound water molecules. We find that the high charge densities of Mg^2+^ and of all phosphates have a strong ordering effect on water, leading to highly structured waters that are visible as high water density. Figure 5a shows a large number of structured water molecules next to the phosphates of ATP and Mg^2+^, while water is much less ordered at the signature motif. The presence of HPO_4_^2−^ bound to the signature motif (Fig. 5b) leads to a number of structured water molecules in its first hydration shell surrounding the signature sequence.

Accordingly, water displacement (average movement of water molecules between consecutive trajectory frames) is strongly affected by the nucleotides. While water displacement in the apo NBD dimer (Fig. 5c) is typical for the first water layer surrounding proteins, the presence of nucleotides increases the number of quasi-bound waters in the interface. Consistent with the bound water structure, displacement of water molecules is reduced next to Mg^2+^ and the phosphates of ATP (Fig. 5d). Displacement of water becomes strongly reduced by the presence of HPO_4_^2−^ (Fig. 5e), as water molecules next to the signature motif become immobile. Importantly, the region of structured water molecules stretches from the signature sequence to the contralateral Walker A motif. Stably bound water molecules establish strong enthalpic interactions, but they also have stringent geometric requirements to form a network of hydrogen bonds and of space, thereby contributing to NBD separation.

**Figure 6:**
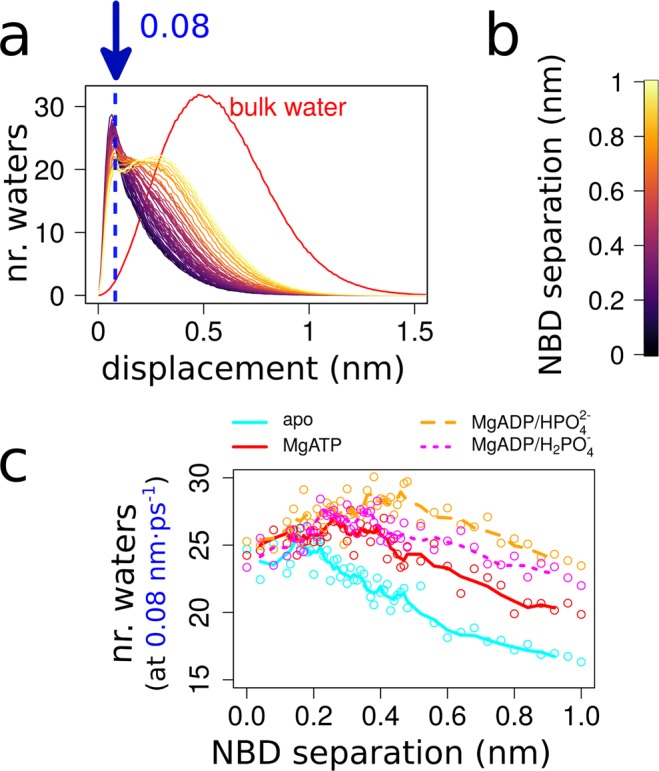
Water accumulating in the NBD interface. (**a**) Number of immobile water molecules between the NBD-NBD interface as a function of NBD separation of the MgATP bound NBD dimer. (**b**) Color scale identifying the NBD separation. (**c**) Distribution of water molecule displacements between consecutive frames as a function of NBD separation.

We quantified the number of stably bound water molecules between the NBDs. Figure 6a shows for the MgATP bound NBDs a series of distributions for the number of water molecules as a function of displacement between consecutive frames per NBD separation (see Supporting Information Fig. 2 for plots for all four systems). For comparison, the distribution of displacements of freely diffusing water molecules is shows in red. In the closed NBD dimer, water molecules in the interface are in contact with the NBDs and show limited displacement. Expanding on the peak at the displacement of 0.08 nm (Fig. 6b), which represents bound water molecules, reveals that the number of structured water molecules is not a monotonic or linear function of NBD separation. For the apo system, the fraction of bound water molecules decreases once NBD separation becomes larger than ~0.2 nm, approximately correlating with the diameter of individual water molecules. The presence of Mg^2+^ + nucleotide + P_i_ in the NBD interface changes the shape of the profile. In line with the larger number of structured water molecules next to the Walker A motif and the signature sequence (Fig. 5), the number of structured water molecules first rises with increasing NBD separation. While the peak is located at ~0.3 nm separation in the presence of MgATP, the peak position of MgADP·HPO_4_^2−^ system is at ~0.5 nm. The position of the peak coincides with the distance at which the enthalpic interaction energy between Mg^2+^ + nucleotide + P_i_ and solvent reaches the plateau value (Fig. 4b).

## Discussion

Substrate transport by ABC transporters is energized by ATP, conformations and conformational changes of the transport cycle are controlled by the ATP-hydrolyzing NBDs. Their motions are associated with MgATP binding, ATP hydrolysis and the release of the hydrolysis products. Unidirectionality of the transport cycle implies the presence of downhill free energy gradients and insurmountable energy barriers. Here we quantified the free energy profile associated with NBD dissociation in the presence of MgATP or the ATP hydrolysis products MgADP·P_i_. The free energy differences of PMF profiles represent the maximal energy output from the NBDs that is available (i) for inducing conformational changes of the TMDs, (ii) for pushing against the lateral pressure of the membrane to accommodate shape changes, (iii) for binding, translocating and releasing substrates, (iv) for adapting the number of water molecules in the central cavity in response to shape changes, (v) and for exchanging MgADP for MgATP.

The free energy profiles (Fig. 3) reveal a clear pattern. NBD dissociation of the apo system shows a very weak stabilization by NBD association with a wide (0.2 nm) and essentially flat energy minimum. The extended flat region implies that association of apo NBDs does not result in a single well defined geometry, consistent with experimental data that could not observe NBD dimers in the absence of nucleotides^60,61^. Binding of MgATP to the NBDs leads to marked change in the free energy profile. The PMF profile shows a narrow and deep energy minimum (−41.8 ± 4.6 kJ/mol), consistent with a single conformation. The steep drop in profile shows that strong forces promote NBD association in the presence of MgATP, but also prevent NBD dissociation without ATP hydrolysis. Consistent with the PMF profile, it is well established that binding of MgATP stabilizes the NBD-dimer^34,35^. Hydrolysis of ATP by the NBSs changes the chemistry of the nucleotides bound to the NBSs. This change destabilizes the closed MgADP·HPO_4_^2−^ bound NBD dimer by inverting the gradient of the PMF profile. The flat profile of the early onset of NBD separation shows that the initial process of NBD separation is slow, followed by a steep gradient exerting strong forces pushing the NBDs apart and representing a barrier for re-association. MD studies^62–64^, where one or both ATPs were replaced by MgADP·P_i_, showed structural changes that were either very subtle or had a stochastic onset. The conformational trapping experiments routinely used to detect the post-hydrolytic state block the transport cycle after P_i_ release, but before ADP dissociation. These vanadate blocking experiments would be unfeasible, if the NBDs would immediately separate^65–67^. Consistently, structural data of the vanadate bound state show that the NBDs only open marginally^68,69^ and vanadate takes the position of P_i_, while MgADP remains bound to the Walker A and the A-loop.

The PMF profiles measure the free energy change as a function of NBD separation. The shapes of the profiles are directly comparable, while their absolute values (the chemical potential μ) depend on the chemical nature of bound nucleotide (nucleotide + Mg^2+^ + P_i_), leading to a vertical shift of the profiles. ATP binding induces NBD association and the formation of a stable state. Consistently, our data show that MgATP-triggered NBD association is a downhill process. Similarly, opening of the NBD dimer after ATP hydrolysis is a downhill process. Importantly, while the interactions between the NBDs and the hydrolysis products (ADP + Mg^2+^ + P_i_) stabilize the post-hydrolytic complex, analysis of the energetic contributions reveals interactions of water as the main driving force for the NBD separation. These downhill movements along the energy profile change the geometry of the NBD dimer.

Reversibility is possible by changing the concentrations in the solution and it has been observed that high concentrations of MgADP inhibit MgATP binding^59^. Also, the transport of substrates by secondary active transporters or the flow of ions through ion channels can be inverted by changes in concentrations of solutes in the internal and external solution^70^. In contrast, directionality of transport in primary active ABC transporters cannot be inverted. The key difference is the chemical reaction of ATP hydrolysis, which is an almost instantaneous process compared to the time scale of protein motions. It releases the chemical energy stored in the phosphate bonds of ATP and creates a high energy state in the closed NBDs. Preventing ATP hydrolysis by mutation or by a non-hydrolysable ATP analog leads to an arrest of the transport cycle in this state. ATP hydrolysis, in contrast to all other steps of the transport cycle, is an irreversible step and consistently, ABCB1 does not function as an ATP synthase.

Conformational changes of the transport cycle reflect relaxations from high energy states by structural changes that follow existing energy gradients. Consequently, we find two high energy states along the transport cycle: (i) the open NBD dimer after MgATP binding and (ii) the post-hydrolytic closed NBD dimer. Exchange of ATP for ADP is assured in energized cells, because the MgATP concentration is typically an order of magnitude higher as compared to ADP and 10-fold above its K_D_. Therefore, MgATP should immediately bind to any available NBSs and should thus not be the rate limiting step of the transport cycle. It is debated^9^, (i) if ATP hydrolysis is needed for reaching the outward facing and substrate-releasing state or (ii) if MgATP-induced NBD dimerisation alone leads to the outward-facing and substrate-releasing state. Experiments performed in whole cells^23^, along with crystal structures, EPR^71^ and single molecule fluorescence studies^72^ indicate that ATP binding shifts ABCB1 to its outward facing state. The affinity for substrates is lowest in the pre-hydrolytic ATP bound state, increases 2 fold in the post-hydrolytic vanadate trapped state, and 5 fold in the MgADP bound state after the release of P_i_ and is highest in the absence of nucleotides^73,74^, indicating that the TMDs can assume more than two conformations. Also, cysteine cross-linking studies showed that both binding and hydrolysis of ATP change the conformation of the TMDs^75^. Consistently, our data on MgATP-induced NBD dimerization and MgADP + P_i_ triggered NBD separation indicate large energy output by the NBDs (−41.8 ± 4.6 kJ/mol and −24.4 ± 4.7 kJ/mol, respectively), which is available for inducing changes in TMD conformation.

## Conclusions

Our PMF calculations show that the transport cycle of ABCB1 includes at least two separated events of energy input that promote progression of ABCB1 through the transport cycle. The first event is provided by ATP binding to the nucleotide-free transporter with separated NBDs, which is of high energy compared to the MgATP-bound closed NBD dimer, therefore leading to NBD dimerisation. The second energy input is ATP hydrolysis, a pseudo-instantaneous change compared to the structural dynamics of ABCB1. The initial confinement of the ATP hydrolysis products MgADP + P_i_ is of high energy and their strong interactions with water leads to NBD separation. Together, these two energy input events drive the catalytic cycle of ABCB1 and make the transport cycle irreversible.

## Supplementary information

Supplementary Information.
