# Supplementary material for: Conversion of chemical to mechanical energy by the nucleotide binding domains of ABCB1
Source: Sci Rep. 2020 Feb 13;10:2589. doi: 10.1038/s41598-020-59403-7 (PMC7018802; doi:10.1038/s41598-020-59403-7)
Supplement: Supplementary file 1 — Supplementary Information. [file 41598_2020_59403_MOESM1_ESM.pdf]

Supplementary Information

Title:
Conversion of chemical to mechanical energy by the nucleotide binding domains of ABCB1

Authors:
Dániel Szöllősi[1], Peter Chiba[2], Gergely Szakacs[3], and Thomas Stockner[1]*

Affiliations:
[1]Medical University of Vienna, Center for Physiology and Pharmacology, Institute of Pharmacology, Waehringerstr. 13A, 1090 Vienna, Austria
[2]Medical University of Vienna, Institute of Medical Chemistry, Center for Pathobiochemistry and Genetics, Waehringerstr. 10, 1090 Vienna, Austria
[3]Medical University of Vienna, Institute of Cancer Research, Borschkegasse 8A, Vienna 1090, Austria

**Corresponding Author:*
Thomas Stockner
Email: thomas.stockner@meduniwien.ac.at

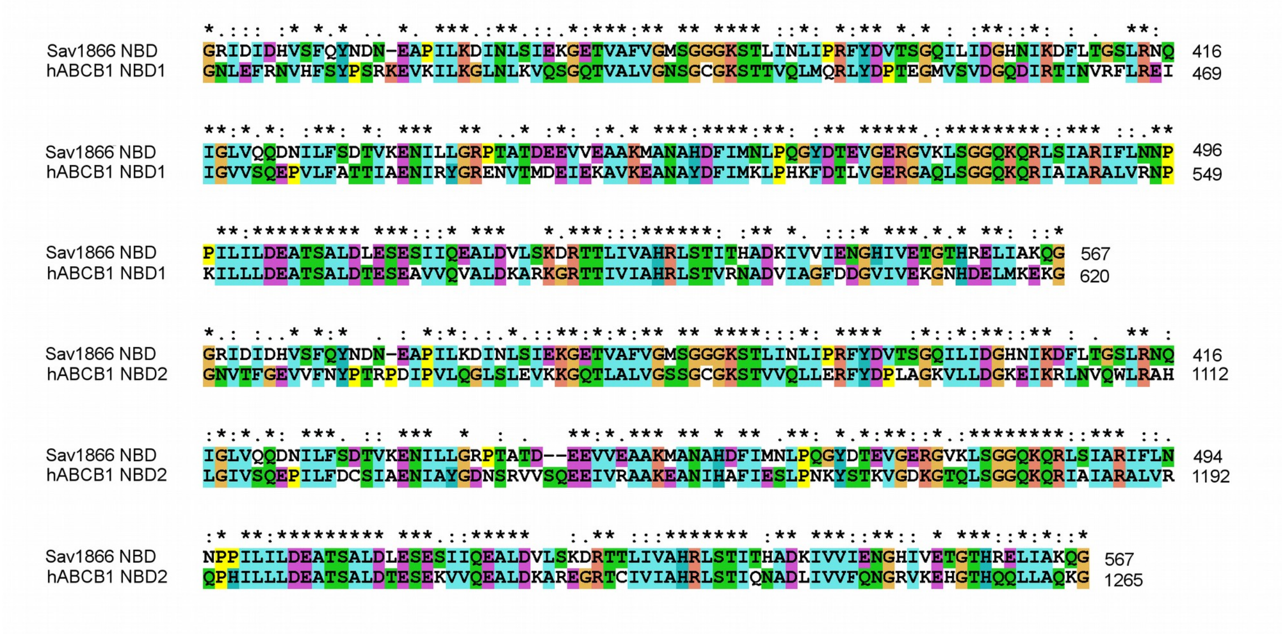

Supplementary Fig. 1: Sequence alignment: Sequence alignment of Sav1866 NBD (residue number 338-567) with the NBD1 (390-620) and NBD2 (1033-1265) of ABCB1. Consensus symbols: ‘*’ - fully conserved residue; ‘:’ strongly similar properties (scoring > 0.5 in the Gonnet PAM 250 matrix) ‘.’ weakly similar properties (scoring =< 0.5 in the Gonnet PAM 250 matrix)

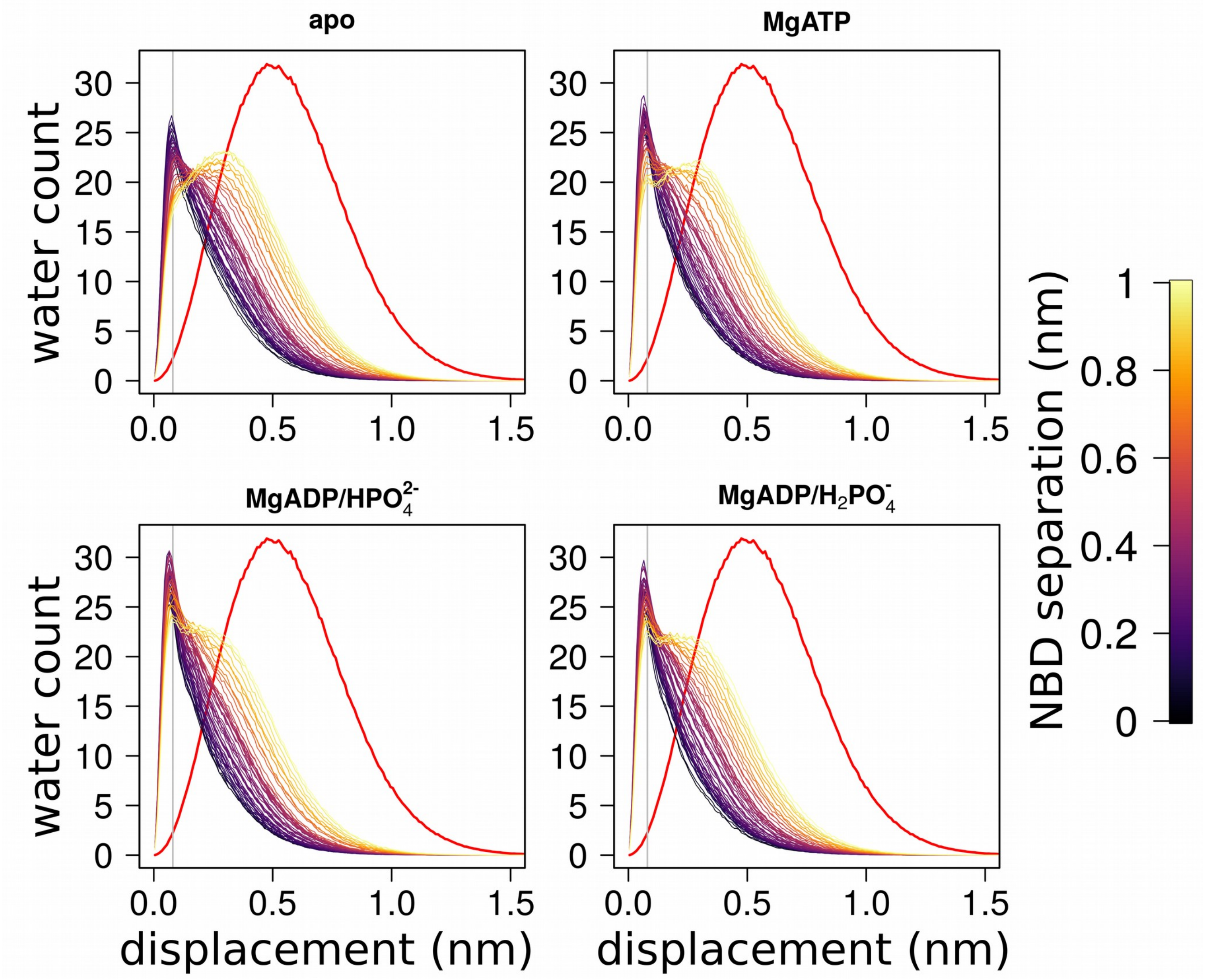

Supplementary Fig. 2:. Distribution of average water displacements. The mean displacement is quantified as the average movement of water molecules between two consecutive frames, separated by 10 ps. Water molecule selection was updated every frame, using the box shown in Supplementary Fig. 3. The corresponding NBD-NBD separation is color coded and shown in the legend to the right. The red distribution corresponds to the averaged water displacements observed for unperturbed water at a large distance from the NBDs. The vertical gray line at 0.08 nm reflects the displacement of structural water molecules due to thermal motions.

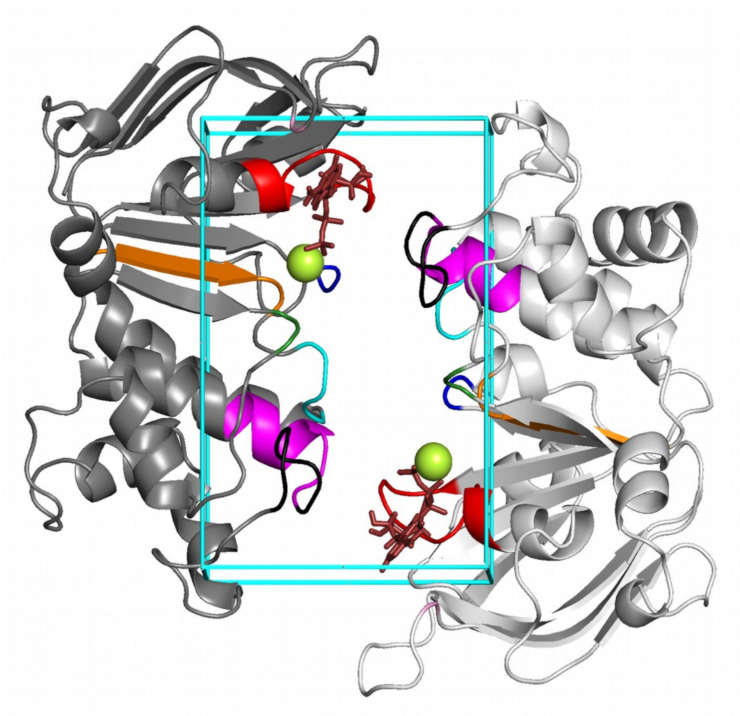

Supplementary Fig. 3: Volume box. Water molecules within the cyan box are considered for the water displacement analysis.

Parameter file used as input for umbrella simulations

```
; VARIOUS PREPROCESSING OPTIONS
; Preprocessor information: use cpp syntax.
; e.g.: -I/home/joe/doe -I/home/mary/roe
;include                   =
; e.g.: -DPOSRES -DFLEXIBLE (note these variable names are case sensitive)
;define                    = -DPOSRES

; RUN CONTROL PARAMETERS
integrator               = md
; Start time and timestep in ps
tinit                    = 0
dt                       = 0.002
nsteps                   = 5000000
; For exact run continuation or redoing part of a run
init_step                = 0
; Part index is updated automatically on checkpointing (keeps files separate)
simulation_part          = 1
; mode for center of mass motion removal
comm-mode                = linear
; number of steps for center of mass motion removal
nstcomm                  = 100
; group(s) for center of mass motion removal
comm-grps                = system

; LANGEVIN DYNAMICS OPTIONS
; Friction coefficient (amu/ps) and random seed
bd-fric                  = 0
ld-seed                  = 1993

; ENERGY MINIMIZATION OPTIONS
; Force tolerance and initial step-size
emtol                    = 1000
emstep                   = 0.0001
; Max number of iterations in relax-shells
niter                    = 20
; Step size (ps^2) for minimization of flexible constraints
fcstep                   = 0.001
; Frequency of steepest descents steps when doing CG
nstcgsteep               = 50
nbfgscorr                = 100

; TEST PARTICLE INSERTION OPTIONS
rtpi                     = 0.05

; OUTPUT CONTROL OPTIONS
; Output frequency for coords (x), velocities (v) and forces (f)
nstxout                  = 5000
nstvout                  = 5000
nstfout                  = 0
; Output frequency for energies to log file and energy file
nstlog                   = 1000
nstcalcenergy            = 100
nstenergy                = 1000
; Output frequency and precision for .xtc file
nstxout-compressed       = 100000
compressed-x-precision   = 1000
; This selects the subset of atoms for the compressed
; trajectory file. You can select multiple groups. By
; default, all atoms will be written.
compressed-x-grps        =
; Selection of energy groups
energygrps               = Protein

; NEIGHBORSEARCHING PARAMETERS
```

```
; cut-off scheme (Verlet: particle based cut-offs, group: using charge groups)
cutoff-scheme            = Verlet
; nblist update frequency
nstlist                  = 20
; ns algorithm (simple or grid)
ns-type                  = Grid
; Periodic boundary conditions: xyz, no, xy
pbc                      = xyz
periodic_molecules       = no
; Allowed energy error due to the Verlet buffer in kJ/mol/ps per atom,
; a value of -1 means: use rlist
verlet-buffer-tolerance  = 0.005
; nblist cut-off
rlist                    = 0.9
; long-range cut-off for switched potentials
rlistlong                = -1
nstcalclr                = -1

; OPTIONS FOR ELECTROSTATICS AND VDW
; Method for doing electrostatics
coulombtype              = PME
coulomb-modifier         = Potential-shift-Verlet
rcoulomb-switch          =
rcoulomb                 = 0.9
; Relative dielectric constant for the medium and the reaction field
epsilon_r                = 1.0
epsilon_rf               = 1
; Method for doing Van der Waals
vdw-type                 = Cut-off
vdw-modifier             = Potential-shift-Verlet
; cut-off lengths
rvdw-switch              =
rvdw                     = 0.9
; Apply long range dispersion corrections for Energy and Pressure
DispCorr                 = EnerPres
; Extension of the potential lookup tables beyond the cut-off
table-extension          = 1
; Separate tables between energy group pairs
energygrp-table          =
; Spacing for the PME/PPPM FFT grid
fourierspacing           = 0.16
; FFT grid size, when a value is 0 fourierspacing will be used
fourier_nx               = 0
fourier_ny               = 0
fourier_nz               = 0
; EWALD/PME/PPPM parameters
pme_order                = 6
ewald_rtol               = 1e-05
ewald-rtol-lj            = 0.001
lj-pme-comb-rule         = Geometric
ewald_geometry           = 3d
epsilon_surface          = 0

; IMPLICIT SOLVENT ALGORITHM
implicit_solvent         = No

; GENERALIZED BORN ELECTROSTATICS
; Algorithm for calculating Born radii
gb-algorithm             = Still
; Frequency of calculating the Born radii inside rlist
nstgbradii               = 1
; Cutoff for Born radii calculation; the contribution from atoms
; between rlist and rgbradii is updated every nstlist steps
rgbradii                 = 1
; Dielectric coefficient of the implicit solvent
gb-epsilon-solvent       = 80
; Salt concentration in M for Generalized Born models
```

```
gb-saltconc              = 0
; Scaling factors used in the OBC GB model. Default values are OBC(II)
gb-obc-alpha             = 1
gb-obc-beta              = 0.8
gb-obc-gamma             = 4.85
gb-dielectric-offset     = 0.009
sa-algorithm             = Ace-approximation
; Surface tension (kJ/mol/nm^2) for the SA (nonpolar surface) part of GBSA
; The value -1 will set default value for Still/HCT/OBC GB-models.
sa-surface-tension       = -1

; OPTIONS FOR WEAK COUPLING ALGORITHMS
; Temperature coupling
tcoupl                   = v-rescale
nsttcouple               = -1
nh-chain-length          = 10
print-nose-hoover-chain-variables = no
; Groups to couple separately
tc-grps                  = Protein non-Protein
; Time constant (ps) and reference temperature (K)
tau-t                    = 0.5   0.5
ref-t                    = 310   310
; pressure coupling
Pcoupl                   = Parrinello-Rahman ; Berendsen for EM and equilibration;Parrinello-Rahman
for production
Pcoupltype               = isotropic
nstpcouple               = -1
; Time constant (ps), compressibility (1/bar) and reference P (bar)
tau-p                    = 20.1; Use 5 for Berendsen; 20 for Parrinello-Rahman;
compressibility          = 4.5e-05   4.5e-05
ref-p                    = 1.0        1.0
; Scaling of reference coordinates, No, All or COM
refcoord_scaling         = All

; OPTIONS FOR QMMM calculations
QMMM                     = no
; Groups treated Quantum Mechanically
QMMM-grps                =
; QM method
QMmethod                 =
; QMMM scheme
QMMMscheme               = normal
; QM basisset
QMbasis                  =
; QM charge
QMcharge                 =
; QM multiplicity
QMmult                   =
; Surface Hopping
SH                       =
; CAS space options
CASorbitals              =
CASelectrons             =
SAon                     =
SAoff                    =
SAsteps                  =
; Scale factor for MM charges
MMChargeScaleFactor      = 1
; Optimization of QM subsystem
bOPT                     =
bTS                      =

; SIMULATED ANNEALING
; Type of annealing for each temperature group (no/single/periodic)
annealing                = no
; Number of time points to use for specifying annealing in each group
annealing-npoints        =
```

```
; List of times at the annealing points for each group
annealing-time           =
; Temp. at each annealing point, for each group.
annealing-temp           =

; GENERATE VELOCITIES FOR STARTUP RUN
gen-vel                  = no
gen-temp                 = 310.0
gen-seed                 = -1

; OPTIONS FOR BONDS
constraints              = all-bonds
; Type of constraint algorithm
constraint-algorithm     = lincs
; Do not constrain the start configuration
continuation             = no
; Use successive overrelaxation to reduce the number of shake iterations
Shake-SOR                = yes
; Relative tolerance of shake
shake-tol                = 0.0001
; Highest order in the expansion of the constraint coupling matrix
lincs-order              = 4
; Number of iterations in the final step of LINCS. 1 is fine for
; normal simulations, but use 2 to conserve energy in NVE runs.
; For energy minimization with constraints it should be 4 to 8.
lincs-iter               = 2
; Lincs will write a warning to the stderr if in one step a bond
; rotates over more degrees than
lincs-warnangle          = 30
; Convert harmonic bonds to morse potentials
morse                    = no

; ENERGY GROUP EXCLUSIONS
; Pairs of energy groups for which all non-bonded interactions are excluded
energygrp-excl           =

; WALLS
; Number of walls, type, atom types, densities and box-z scale factor for Ewald
nwall                    = 0
wall_type                = 9-3
wall_r_linpot            = -1
wall-atomtype            =
wall-density             =
wall_ewald_zfac          = 3

;---------------------------------------------------------------------------

; COM PULLING
pull                     = yes
; Cylinder radius for dynamic reaction force groups (nm)
pull-cylinder-r          = 1.5
pull_constr_tol          = 1e-06
pull-print-com1          = no
pull-print-com2          = no
pull-print-ref-value     = no
pull-print-components    = no
pull_nstxout             = 100
pull_nstfout             = 100
; Number of pull groups
pull-ngroups             = 2
; Number of pull coordinates
pull-ncoords             = 1
; Group name, weight (default all 1), vector, init, rate (nm/ps), kJ/(mol*nm^2)
pull-group1-name         = Chain_A
pull-group1-weights      =
pull-group1-pbcatom      = 2688
pull-group2-name         = Chain_B
```

```
pull-group2-weights      =
pull-group2-pbcatom      = 6306
pull_coord1_type         = umbrella
pull_coord1_geometry     = direction
pull-coord1-groups       = 1 2
pull-coord1-dim          = Y N N
pull-coord1-origin       = 0.0 0.0 0.0
pull_coord1_vec          = 1.0 0.0 0.0
pull-coord1-start        = yes
;pull-coord1-init         = 0
pull_coord1_rate         = 0.0
pull_coord1_k            = 5000
pull_coord1_kB           = 0

;-----------------------------------------------------------------------------

; ENFORCED ROTATION
; Enforced rotation: No or Yes
rotation                 = yes
; Output frequency for angle, torque and rotation potential energy for the whole group
rot_nstrout              = 100
; Output frequency for per-slab data (angles, torques and slab centers)
rot-nstsout              = 1000
; Number of rotation groups
rot_ngroups              = 6


rot_group0               = Chain_A
rot_type0                = rm2
rot_massw0               = no
rot_vec0                 = 1.0 0.0 0.0
rot-pivot0               = 4.626513671875 3.9065689086914066 3.74798698425293
rot_rate0                = 0
rot_k0                   = 1000
rot-slab-dist0           = 1.5
rot-min-gauss0           = 0.001
rot-eps0                 = 0.01
rot_fit_method0          = rmsd
rot_potfit_nsteps0       = 21
rot_potfit_step0         = 0.25

rot_group1               = Chain_A
rot_type1                = rm2-pf
rot_massw1               = no
rot_vec1                 = 0.0 1.0 0.0
rot-pivot1               = 4.626513671875 3.9065689086914066 3.74798698425293
rot_rate1                = 0
rot_k1                   = 1000
rot-slab-dist1           = 1.5
rot-min-gauss1           = 0.001
rot-eps1                 = 0.01
rot_fit_method1          = rmsd
rot_potfit_nsteps1       = 21
rot_potfit_step1         = 0.25

rot_group2               = Chain_A
rot_type2                = rm2-pf
rot_massw2               = no
rot_vec2                 = 0.0 0.0 1.0
rot-pivot2               = 4.626513671875 3.9065689086914066 3.74798698425293
rot_rate2                = 0
rot_k2                   = 1000
rot-slab-dist2           = 1.5
rot-min-gauss2           = 0.001
rot-eps2                 = 0.01
rot_fit_method2          = rmsd
rot_potfit_nsteps2       = 21
```

```
rot_potfit_step2         = 0.25

; Rotation group name
rot_group3               = Chain_B
; Rotation potential. Can be iso, iso-pf, pm, pm-pf, rm, rm-pf, rm2, rm2-pf, flex, flex-t, flex2,
flex2-t
rot_type3                = rm2
; Use mass-weighting of the rotation group positions
rot_massw3               = no
; Rotation vector, will get normalized
rot_vec3                 = 1.0 0.0 0.0
; Pivot point for the potentials iso, pm, rm, and rm2 (nm)
rot-pivot3                = 7.117191314697266 5.076591491699219 3.7540428161621096 ;
; Rotation rate (degree/ps) and force constant (kJ/(mol*nm^2))
rot_rate3                = 0
rot_k3                   = 1000
; Slab distance for flexible axis rotation (nm)
rot-slab-dist3           = 1.5
; Minimum value of Gaussian function for the force to be evaluated (for flex* potentials)
rot-min-gauss3           = 0.001
; Value of additive constant epsilon' (nm^2) for rm2* and flex2* potentials
rot-eps3                 = 0.01
; Fitting method to determine angle of rotation group (rmsd, norm, or potential)
rot_fit_method3          = rmsd
; For fit type 'potential', nr. of angles around the reference for which the pot. is evaluated
rot_potfit_nsteps3       = 21
; For fit type 'potential', distance in degrees between two consecutive angles
rot_potfit_step3         = 0.25

rot_group4               = Chain_B
rot_type4                = rm2-pf
rot_massw4               = no
rot_vec4                 = 0.0 1.0 0.0
rot-pivot4               = 7.117191314697266 5.076591491699219 3.7540428161621096
rot_rate4                = 0
rot_k4                   = 1000
rot-slab-dist4           = 1.5
rot-min-gauss4           = 0.001
rot-eps4                 = 0.01
rot_fit_method4          = rmsd
rot_potfit_nsteps4       = 21
rot_potfit_step4         = 0.25

rot_group5               = Chain_B
rot_type5                = rm2-pf
rot_massw5               = no
rot_vec5                 = 0.0 0.0 1.0
rot-pivot5                = 7.117191314697266 5.076591491699219 3.7540428161621096
rot_rate5                = 0
rot_k5                   = 1000
rot-slab-dist5           = 1.5
rot-min-gauss5           = 0.001
rot-eps5                 = 0.01
rot_fit_method5          = rmsd
rot_potfit_nsteps5       = 21
rot_potfit_step5         = 0.25

;-------------------------------------------------------------------

; Group to display and/or manipulate in interactive MD session
IMD-group                =

; NMR refinement stuff
; Distance restraints type: No, Simple or Ensemble
disre                    = No
; Force weighting of pairs in one distance restraint: Conservative or Equal
disre-weighting          = Conservative
```

```
; Use sqrt of the time averaged times the instantaneous violation
disre-mixed              = no
disre-fc                 = 100
disre-tau                = 0
; Output frequency for pair distances to energy file
nstdisreout              = 5000
; Orientation restraints: No or Yes
orire                    = no
; Orientation restraints force constant and tau for time averaging
orire-fc                 = 0
orire-tau                = 0
orire-fitgrp             =
; Output frequency for trace(SD) and S to energy file
nstorireout              = 100

; Free energy variables
free-energy              = no
couple-moltype           =
couple-lambda0           = vdw-q
couple-lambda1           = vdw-q
couple-intramol          = no
init-lambda              = 0
init-lambda-state        = -1
delta-lambda             = 0
nstdhdl                  = 50
fep-lambdas              =
mass-lambdas             =
coul-lambdas             =
vdw-lambdas              =
bonded-lambdas           =
restraint-lambdas        =
temperature-lambdas      =
calc-lambda-neighbors    = 1
init-lambda-weights      =
dhdl-print-energy        = no
sc-alpha                 = 0
sc-power                 = 1
sc-r-power               = 6
sc-sigma                 = 0.3
sc-coul                  = no
separate-dhdl-file       = yes
dhdl-derivatives         = yes
dh_hist_size             = 0
dh_hist_spacing          = 0.1

; Non-equilibrium MD stuff
acc-grps                 =
accelerate               =
freezegrps               =
freezedim                =
cos-acceleration         = 0
deform                   =

; simulated tempering variables
simulated-tempering      = no
simulated-tempering-scaling = geometric
sim-temp-low             = 300
sim-temp-high            = 300

; Electric fields
; Format is number of terms (int) and for all terms an amplitude (real)
; and a phase angle (real)
E-x                      =
; Time dependent (pulsed) electric field. Format is omega, time for pulse
; peak, and sigma (width) for pulse. Sigma = 0 removes pulse, leaving
; the field to be a cosine function.
E-xt                     =
```

```
E-y                      =
E-yt                     =
E-z                      =
E-zt                     =

; Ion/water position swapping for computational electrophysiology setups
; Swap positions along direction: no, X, Y, Z
swapcoords               = no

; AdResS parameters
adress                   = no

; User defined thingies
user1-grps               =
user2-grps               =
userint1                 = 0
userint2                 = 0
userint3                 = 0
userint4                 = 0
userreal1                = 0
userreal2                = 0
userreal3                = 0
userreal4                = 0
```
